# Integrated whole-genome screening for Pseudomonas aeruginosa virulence genes using multiple disease models reveals that pathogenicity is host specific

**DOI:** 10.1111/1462-2920.12863

**Published:** 2015-05-14

**Authors:** Jean-Frédéric Dubern, Cristina Cigana, Maura De Simone, James Lazenby, Mario Juhas, Stephan Schwager, Irene Bianconi, Gerd Döring, Leo Eberl, Paul Williams, Alessandra Bragonzi, Miguel Cámara

**Affiliations:** 1 School of Life Sciences, Centre for Biomolecular Sciences, University of NottinghamNottingham, UK; 2 Division of Immunology, Transplantation and Infectious Diseases, IRCCS, San Raffaele Scientific InstituteMilano, Italy; 3 Department of Microbiology, Institute of Plant Biology, University of ZürichZürich, Switzerland; 4 Institute of Medical Microbiology and Hygiene, University of TübingenTübingen, Germany

## Abstract

*Pseudomonas aeruginosa* is a multi-host opportunistic pathogen causing a wide range of diseases because of the armoury of virulence factors it produces, and it is difficult to eradicate because of its intrinsic resistance to antibiotics. Using an integrated whole-genome approach, we searched for *P*. *aeruginosa* virulence genes with multi-host relevance. We constructed a random library of 57 360 Tn5 mutants in *P*. *aeruginosa* PAO1-L and screened it *in vitro* for those showing pleiotropic effects in virulence phenotypes (reduced swarming, exo-protease and pyocyanin production). A set of these pleiotropic mutants were assayed for reduced toxicity in *Drosophila melanogaster,* *Caenorhabditis elegans*, human cell lines and mice. Surprisingly, the screening revealed that the virulence of the majority of *P*. *aeruginosa* mutants varied between disease models, suggesting that virulence is dependent on the disease model used and hence the host environment. Genomic analysis revealed that these virulence-related genes encoded proteins from almost all functional classes, which were conserved among *P*. *aeruginosa* strains. Thus, we provide strong evidence that although *P*. *aeruginosa* is capable of infecting a wide range of hosts, many of its virulence determinants are host specific. These findings have important implication when searching for novel anti-virulence targets to develop new treatments against *P*. *aeruginosa*.

## Introduction

*Pseudomonas aeruginosa* is adapted to thrive in different environments and is one of the top three causes of opportunistic human infections responsible for causing millions of cases each year in the community and 10–15% of all health care-associated infections (Lyczak *et al*., 2000; Boucher *et al*., 2009). Various body sites may be affected by *P. aeruginosa* infections including the respiratory tract, skin and soft tissues, the urinary tract, post-operative and burn wounds, brain, heart, bloodstream and cornea. Infections caused by this opportunistic pathogen are often life threatening and they are of particular concern for intensive care units where ventilated patients may develop ventilator-associated pneumonia and sepsis (Gellatly and Hancock, 2013). Other patients at risk of acquiring *P. aeruginosa* are those with compromised immune system, due to immunosuppressive therapies or underlying diseases such as cancer, acquired immune deficiency syndrome (AIDS) or the hereditary disease cystic fibrosis (CF).

Antibiotics are used as the first line of action against *P. aeruginosa* infections. However, the frequently observed inefficacy of this type of treatment is linked to the high levels of intrinsic and acquired resistance of *P. aeruginosa* to these therapeutic agents (Poole, 2011). Despite growing evidence about the severity of the diseases caused by *P. aeruginosa*, no drugs with a novel mechanism of action against this organism have reached the market in recent years and all the other registered drugs against Gram-negatives do not cover *Pseudomonas* in their spectrum (Page and Heim, 2009). Thus, there is an urgent need for the discovery of novel alternative strategies to the traditional use of antibiotics to combat *P. aeruginosa* infections. Anti-virulence therapies have become an attractive approach that may yield drugs with high specificity and narrow spectra (Fernebro, 2011). These novel therapies are beginning to change the perspectives on infectious disease control. Instead of reducing pathogen burden directly (‘pathogen elimination’), anti-virulence therapies reduce the illness caused by the pathogen (‘damage limitation’) (Vale *et al*., 2014). From the perspective of the patient, reducing illness is the first priority even before killing the cause.

During the past few years, remarkable progress has been made in the identification of *P. aeruginosa* virulence factors and the characterization of the mechanisms they use to cause disease in the human host and a range of disease models. Major *P. aeruginosa* virulence factors have been identified at the single gene level by classical genetics; that is, by searching for a mutant with a particular phenotype, followed by the identification of the mutated gene and its characterization. Few studies have used a genome-wide analysis through the screening of *P. aeruginosa* mutant libraries (Liberati *et al*., 2006) in different non-vertebrate hosts including *Caenorhabditis elegans* (Feinbaum *et al*., 2012) and *Drosophila melanogaster* (Kim *et al*., 2008). Furthermore, high-throughput screenings using transposon mutants tagged with a unique oligonucleotide (e.g. signature tagged mutagenesis) in vertebrate models including rats and mice have been employed to identify virulence genes involved in different stages of infection (e.g. acute or chronic) (Potvin *et al*., 2003; Winstanley *et al*., 2009; Bianconi *et al*., 2011). Early studies showed that the virulence mechanisms used by *P. aeruginosa* to infect phylogenetically diverse hosts were remarkably well conserved, suggesting that the dissection of these mechanisms in one single model system could provide reliable understanding of the mechanisms used by *P. aeruginosa* to cause disease in mammals (Finlay, 1999). Direct evidence that *P. aeruginosa* uses a shared subset of virulence factors to elicit disease has been provided first comparing the plant *Arabidopsis thaliana* with mice (Rahme *et al*., 1995), and thereafter extending the knowledge to the nematode *C. elegans* (Tan *et al*., 1999), the silkworm *Bombyx mori* (Chieda *et al*., 2005), the insects *Galleria melonella* (Jander *et al*., 2000) and *D. melanogaster* (Apidianakis and Rahme, 2009), the amoeba *Dictyostelium discoideum* (Cosson *et al*., 2002) and the zebrafish embryo (Phennicie *et al*., 2010). Limitations of these studies are related to the restricted number of *P. aeruginosa* strains tested, and the impact of virulence in multi-host system by employing large high-throughput screenings remains to be established.

The sequencing of the first *P. aeruginosa* genome in 2000 revealed that the PAO1 strain sequenced (now PAO1-UW) has a genome size of 6.3 Mbp and contains 5570 predicted open reading frames (ORFs), making it the largest bacterial genome sequenced at that time (Stover *et al*., 2000). The large size of the *P. aeruginosa* genome reflects the numerous and distinct gene families that it contains, which is also a reflection of its ability to adapt to many different environments. This is in contrast to some other large bacterial genomes, whose size reflects gene duplication events rather than greater genetic and functional diversity. Specifically, the *P. aeruginosa* genome contains a disproportionately large number of genes predicted to encode outer membrane proteins involved in adhesion, motility, antibiotic efflux, virulence factor export and environmental sensing by two-component systems (Kung *et al*., 2010). Additionally, consistent with the metabolic versatility of this bacterium, the *P. aeruginosa* genome has a large number of genes encoding transport systems and enzymes involved in nutrient uptake and metabolism. Considering the genetic diversity of the *P. aeruginosa* genome, it is not surprising that it contains one of the highest percentages of predicted regulatory genes (8.4%) of all bacterial genomes. Furthermore, the function of nearly 20% of the *P. aeruginosa* genes has been demonstrated experimentally (http://www.pseudomonas.com). The rest of the *P. aeruginosa* genome has been assigned predicted functions, based on homologies to previously characterized genes, which remain to be demonstrated, with more than 500 genes remaining without annotation. Hence, there is still a large amount of information missing with regard to the mechanisms used by this organism to cause disease and, as consequence, a whole array of anti-virulence targets remains to be discovered within it.

As a first step toward selecting targets for anti-virulence therapy, we constructed and screened a random library of 57 360 PAO1-L Tn5 mutants *in vitro* for pleiotropic effects on virulence-related phenotypes (reduced swarming, exo-protease and pyocyanin production) and for reduced toxicity in a sequential cascade of disease models, including *D. melanogaster*, *C. elegans*, airways cell lines and finally a murine model of acute pneumonia. Based on previous knowledge reported above, it was expected that mutants attenuated in one disease model would also show attenuation in the other models. Surprisingly, the screening revealed that pathogenicity of a set of *P. aeruginosa* mutants behaved very differently in different disease models suggesting that virulence is very much dependent on the disease model used. Thus, we provide evidence that although *P. aeruginosa* is capable of infection of a wide range of hosts, many of the virulence determinants are host specific.

## Results

### Genome-wide screening for *P*. *aeruginosa* virulence-related genes *in vitro* and in *C*. *elegans* and *D*. *melanogaster* disease models

#### Primary screen

**Figure 1:**
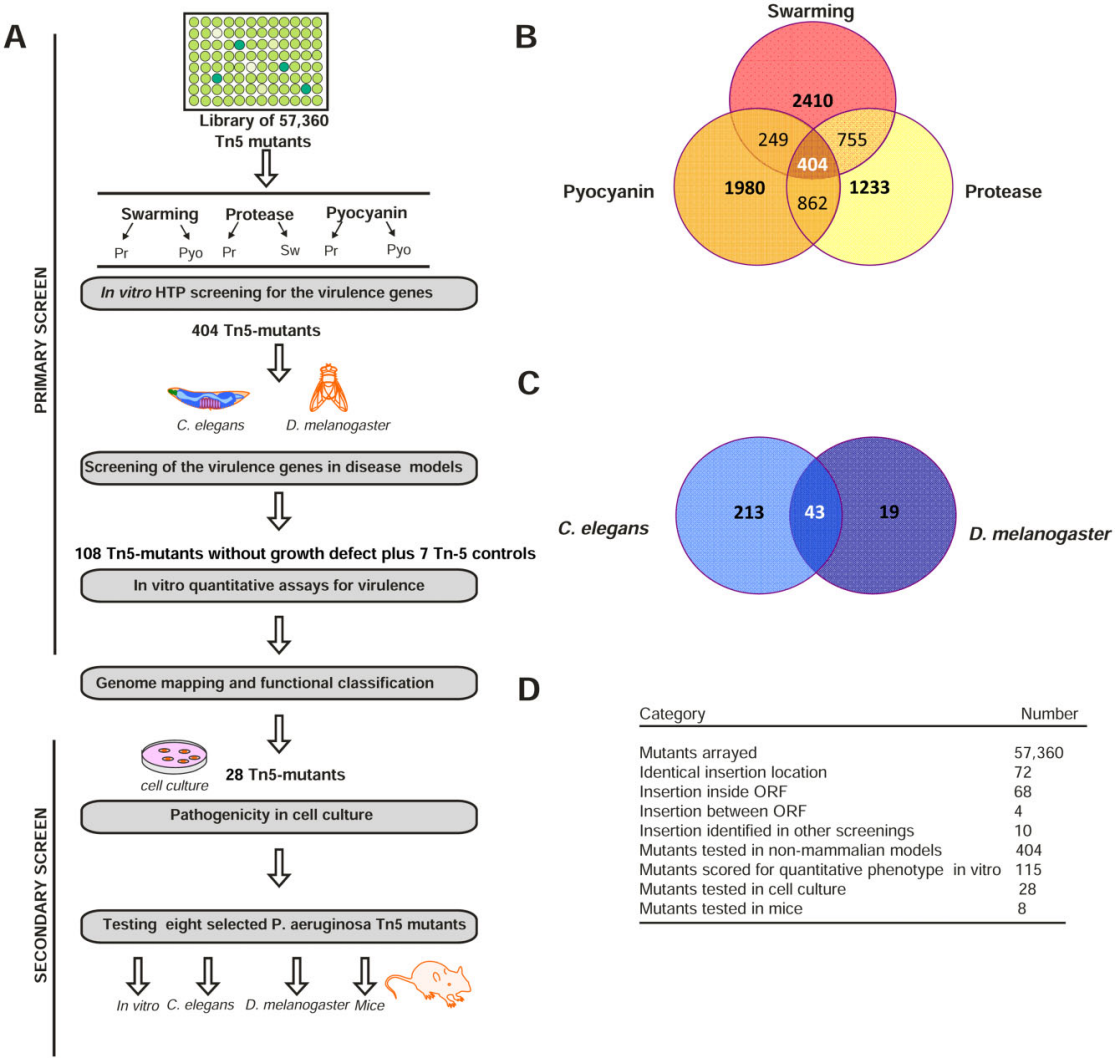
Schematic representation of the Tn5 mutants screening procedure in multi-host system.A. The whole procedure consisted of the following steps, with the remaining number of candidates shown after each step: size of transposon library tested (57 360 Tn5 mutants), primary screening (404 Tn5 mutants selected from *in vitro* screening and 275 from *C*. *elegans* and *D*. *melanogaster* screening), secondary screening (28 Tn5 mutants selected for cell culture models). The final 8 candidates were tested for virulence attenuation in a mouse pneumonia model.B. Summary of the results of the *in vitro* screening. A total of 404 Tn5 mutants having pleiotropic phenotypes (impaired in protease, pyocyanin and swarming motility) were selected from the primary screening and further tested in *C*. *elegans* and *D*. *melanogaster* disease models (C).D. Summary of the results of the transposon library.

The screening for virulence genes in *P. aeruginosa* started with the construction of a Tn5 mutant library in PAO1-L using pLM1 due to the high transposition efficiency of this Tn5-based vector in *P. aeruginosa* (Larsen *et al*., 2002) (Table S1). The overall screening strategy is shown in Fig. 1A. A total of 57 360 Tn5 mutant strains (598 plates with 96 individual mutants each), representing an estimated of 95% of the genome (Liberati *et al*., 2006), were obtained by conjugation. A high-throughput (HTP) screening using a robotic system and suitable bioassays for the Tn5 mutants with attenuated virulence were designed. Due to the large number of mutants to be screened, the primary screening was not done quantitatively for practical reasons. Thus, Tn5 mutants were tested individually in a qualitative manner for attenuation in swarming, the loss of the blue pigment pyocyanin in liquid media and the reduction in protease production using 5% skimmed milk agar plates. A total of 5623 Tn5 mutants with alterations in one of these three phenotypes were subsequently re-screened for attenuation in the other two phenotypes (Fig. 1B). A total of 404 putative virulence-related Tn5 mutants showing pleiotropic effects on the above phenotypes (Fig. 1B) were selected for screening using the *C. elegans* and *D. melanogaster* infection models. This approach enabled further short listing of the Tn5 mutants prior to identifying any redundancies and evaluating them in quantitative disease assays. A total of 232 Tn5 mutants out of the 404 above were found to be attenuated in at least one of the non-vertebrate disease models used, whereas 43 were attenuated in both models (Fig. 1C). Interestingly, in the *D. melanogaster* disease model, fewer mutants (19) showed attenuation compared with *C. elegans* (213). The 275 attenuated mutants were further screened to identify those showing growth differences in relation to the parental strain and which could in part be responsible for the attenuation observed. From this screening, only 108 mutants showed no growth defects (data not shown) and hence were selected further investigation. As part of the validation of the screening strategy used, seven mutants found attenuated in the initial HTP screening, but not in *C. elegans* and *D. melanogaster*, were also included (later found to have Tn5 insertions in *mexA*, *pauB1*, *tgpA*, PA3448, PA3613, *tufA* and PA5156), hence the 115 mutants recorded in Fig. 1A. These control mutants together with the remaining 108 mutants (of which 3 and 62 were only attenuated in *D. melanogaster* and *C. elegans*, respectively, whereas 43 in both models) were tested for alterations in swarming motility, and the production of pyocyanin and alkaline protease. Although the primary qualitative assay by HTP screening showed that all the selected 115 putative virulence-related mutants were attenuated in the three phenotypes, this secondary quantitative assays showed that only 47 of them were attenuated in all three virulence traits, 52 in two traits and 16 in one trait (data not shown). These differences could be attributed to the intrinsic nature of the assays used, from qualitative to quantitative, which may have resulted in changes in certain variables such as oxygen and nutrient availability.

### Genome mapping, functional classification of *P*. *aeruginosa* virulence-related genes and their conservation by comparative genomic analysis

The exact Tn5 insertion site in the PAO1-L genome for each of the 115 putative virulence-related mutants was determined by sequencing the Tn5 flanking genomic regions. These resulted in the mapping of the mutations as well as the identification of some redundancies, which were excluded from further screening. Single insertions within a specific gene in 68 Tn5 mutants, representing 1.3% of the entire genome coding sequences (Stover *et al*., 2000), and 4 single insertion in intergenic regions (PA1030.1, PA4767-68, PA5202-03, PA5360-61) were found (Fig. 1D). Table 1 shows the detailed results of the 72 Tn5 mutants selected, including also 7 mutants without attenuation in non-mammalian models as described before.

**Table 1:** List of 72 Tn5 *P*. *aeruginosa* selected as virulence-related genes

			*In vitro* phenotypic analysis			Non-mammalian models		Cell culture		Murine model	
PA number	Gene name	Functional class	Protease production	Swarming motility	Pyocyanin production	*C. elegans*	*D. melanogaster*	Invasion in A549	IL-8 production in A549	Acute murine lung infection	Identification in other large-scale screenings or annotated in Virulence Factors Database
PA0082	tssA1	Protein secretion/export apparatus	+	+	+	+	−				(Potvin *et al*., 2003), Y
PA0337	ptsP	Transport of small molecules	+	−	+	+	−				(Feinbaum *et al*., 2012)
PA0366		Putative enzymes	−	+	+	+	−				
PA0396	pilU	Motility and attachment	+	−	+	+	−				Y
PA0425	mexA	Transport of small molecules	+	+	+	−	−				
PA0534	pauB1	Carbon compound catabolism	+	+	+	−	−				
PA0593	pdxA	Biosynthesis of cofactors, prosthetic groups and carriers	+	+	+	+	+				
PA0823		Unknown	+	−	+	+	+				
PA0844	plcH	Secreted factors	+	−	+	+	−				Y
PA0914		Unknown	+	−	+	+	−	+	+		
PA0996	pqsA	Biosynthesis of cofactors, prosthetic groups and carriers	+	−	+	+	−	−	−		
PA0998	pqsC	Biosynthesis of cofactors, prosthetic groups and carriers	+	−	+	+	+				
PA1030.1			+	+	+	+	−				
PA1118		Membrane proteins	+	+	+	+	−				
PA1523	xdhB	Nucleotide biosynthesis and metabolism	+	−	−	+	−				
PA1542		Unknown	+	−	+	+	−				
PA1634	kdpB	Transport of small molecules	+	+	+	+	+	+	+	−	
PA1799	parR	Transcriptional regulator	+	+	+	+	−	+	+		
PA2130	cupA3	Motility and attachment	+	+	+	+	−				(Winstanley *et al*., 2009)
PA2240	pslJ	Cell wall/LPS/capsule	+	−	+	+	−				
PA2385	pvdQ	Adaptation and protection	+	+	+	+	−	+	+	+	Y
PA2414		Carbon compound catabolism	+	+	+	+	−	+	+	−	
PA2781		Unknown	−	+	+	+	−				
PA2854		Unknown	+	−	+	+	+				
PA2873	tgpA	Unknown	+	−	−	−	−	+	+		
PA2998	nqrB	Energy metabolism	+	−	+	+	−				(Potvin *et al*., 2003)
											(Bianconi *et al*., 2011)
PA3058	pelG	Cell wall/LPS/capsule	+	+	−	+	+	−	+		
PA3071		Unknown	+	+	+	+	−				
PA3101	xcpT	Protein secretion/export apparatus	+	−	+	+	+				
PA3110		Unknown	+	−	+	+	−				(Potvin *et al*., 2003)
PA3139		Amino acid biosynthesis and metabolism	+	−	+	+	+	−	+		
PA3257	prc	Translation, post-translational modification, degradation	+	−	+	+	−				
PA3270		Unknown	+	+	+	+	−				
PA3327		Adaptation, protection	+	+	+	+	−				
PA3448		Transport of small molecules	+	+	+	−	−				
PA3449		Unknown	+	+	−	−	+	+	+		
PA3460		Putative enzymes	+	+	+	+	+	−	−		
PA3493		Unknown	+	−	+	+	−				
PA3613		Unknown	−	+	+	−	−	−	−	−	
PA3622	rpoS	Transcriptional regulators	+	−	−	+	+	+	+		
PA3649	mucP	Unknown	+	+	−	+	−	+	+		Y
PA3761	nagE	Transport of small molecules	+	−	−	+	+	−	+		
PA3799		Unknown	+	−	+	+	+	+	+		
PA3867		DNA replication, recombination, modification and repair	+	−	+	+	+	+	+		
PA3950		Transcription, RNA processing and degradation	+	−	−	+	−				
PA4000	rlpA	Unknown	+	+	+	+	−				
PA4059		Unknown	+	+	−	+	−	+	+		
PA4098		Putative enzymes	+	−	−	+	+				(Winstanley *et al*., 2009)
PA4113		Transport of small molecules	+	+	+	+	−				
PA4116	bphO	Putative enzymes	+	−	+	+	−	+	+	+	
PA4265	tufA	Translation, post-translational modification, degradation	+	+	−	−	−	−	−		
PA4282		DNA replication, recombination, modification and repair	+	−	+	+	−				
PA4352		unknown	+	+	+	+	−	−	+		
PA4448	hisD	Amino acid biosynthesis and metabolism	+	−	−	−	+				
PA4489	magD	Hypothetical protein	+	−	+	+	−	+	+		(Potvin *et al*., 2003)
PA4498		Translation, post-translational modification, degradation	−	+	+	+	+				
PA4515		Unknown	+	+	+	+	+				
PA4528	pilD	Motility & Attachment	+	+	+	+	−				(Potvin *et al*., 2003), Y
PA4684		Unknown	+	−	+	+	−				
PA4767-68			−	+	+	+	+	+	−		
PA4768	smpB	Translational, post-translational modification, degradation	−	+	+	+	−	−	+		
PA4809	fdhE	Energy metabolism	+	+	−	+	−				
PA4916		Unknown	+	+	+	−	+	+	−	+	
PA5022		Unknown	+	−	+	+	−				
PA5138		Unknown	+	+	+	+	−				
PA5156		Unknown	+	+	+	−	−	+	+	+	
PA5202-03			+	+	+	+	+				
PA5203	gshA	Amino acid biosynthesis and metabolism/cofactors, prosthetic group	+	+	+	+	−	+	+		(Feinbaum *et al*., 2012)
PA5332	crc	Energy metabolism	+	+	−	+	+	+	+	+	(Liberati *et al*., 2006)
PA5360-61	phoB-phoR		+	+	+	+	−				
PA5361	phoR	Transcriptional regulators	+	−	+	+	−				
PA5548		Transport of small molecules	+	+	+	+	−				

+ attenuated in comparison with wild-type PAO1;

− not attenuated in comparison with wild-type PAO1; LPS, lipopolysaccharide; Nd: not determined; Y, present in the Virulence Factor Database (http://www.mgc.ac.cn/VFs/).

The 68 ORFs identified with Tn5 insertions encoded proteins from almost all functional classes (Fig. S1): hypothetical, unknown, unclassified proteins (25 genes), transport of small molecules (7), carbon metabolism and energy metabolism (5), translation and post-transcriptional modification (4), amino acid metabolism (3), transcriptional regulators (3), biosynthesis of co-factors and prosthetic group (3), motility and attachment (3), putative enzyme (3), cell wall/lipopolysaccharide (LPS)/capsule (2), secretion and export (2), adaptation and protection (2), DNA replication (2), transcription and RNA process/degradation (1), secreted factors (1), membrane protein (1) and nucleotide biosynthesis and metabolism (1). From all the genes identified, some of them had been previously demonstrated to be virulence determinants in *P. aeruginosa*. The role of these genes included antibiotic resistance (*parR*, *phoR*, *mexA*), biofilm formation (*pelG*, *pslJ*, *mucP* and *cupA3*), virulence factor delivery (*tssA1*, *pilD*, *xcpT* and *plcH*), regulation (*pqsA*, *pqsC*, *pvdQ*, *rpoS, crc*, *pilU* and *ptsP*) and metabolism (*bphO* and *nagE*) (Table 1), supporting the robustness of the screening strategy used.

Next, the presence and level of conservation of the virulence-related genes identified in this study were determined in different *P. aeruginosa* strains and in other bacteria. Using the PAO1-UW genome (http://www.pseudomonas.com) as a reference, a predicted amino acid sequence-based comparative analysis for selected targets was carried out against six *P. aeruginosa*-sequenced genomes (PA14, LESB58, PA7, 2192, C3719 and PACS2). Furthermore, conservation was also investigated against the genomes of *Escherichia coli* K12 and *Burkholderia cenocepacia* J2315 (Table S2). The majority of the selected targets (58) were found conserved in all the six *P. aeruginosa* genomes analysed, seven (PA0914, CupA3, PA3110, PA3327, RpoS, PA4282, PA5548) were conserved in five genomes, one (TufA) in four genomes and two (PA0823 and PA3867) only in three genomes. Ten targets were highly conserved in *B. cenocepacia* J2315, with an identity higher than 60%, whereas 41 had no or poor (< 40%) identity. Analysis against the *E. coli* K12 genome revealed that only five targets were highly conserved, with an identity higher than 60%, whereas 34 have no or poor (< 40%) identity. A homologue of tufA with very high identity (> 80%) at the protein level was found in both *B. cenocepacia* J2315 and *E. coli* K12. The majority of the selected targets showed no clear homologues in humans, suggesting that they may be considered further for anti-virulence therapy.

### Pathogenicity of *P*. *aeruginosa* selected virulence-related genes in human cell culture model

#### Secondary screen

**Figure 2:**
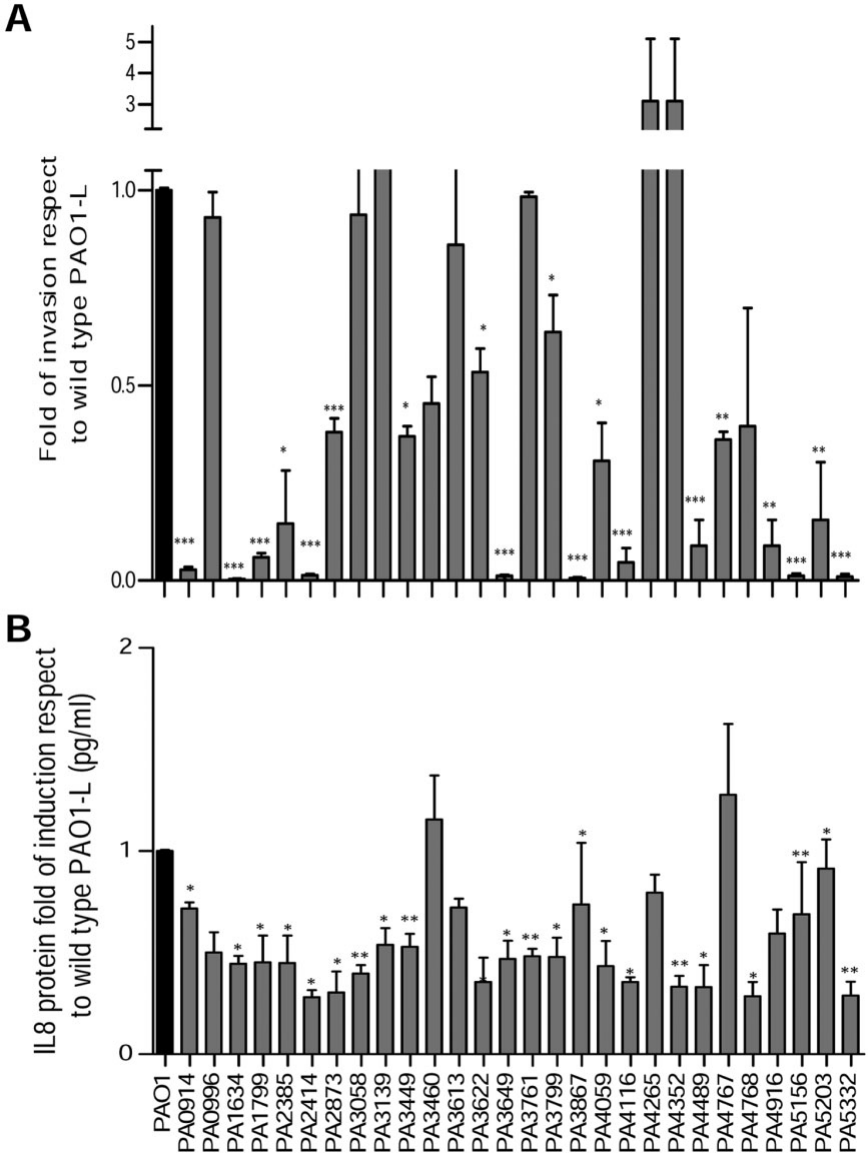
Invasion and IL-8 release in A549 cells of selected Tn-5 mutants.(A) Invasion by antibiotic exclusion assay and (B) IL-8 release by ELISA were determined in A549 cells after infection with wild-type and Tn5 mutants. Data, from three independent experiments, are expressed as mean ± standard error of the mean (SEM). *P < 0.05, **P < 0.01, ***P < 0.001, Student’s *t*-test.

To evaluate whether virulence attenuation found in the *in vitro* and non-mammalian screenings may be confirmed in mammalian cell models, 28 putative virulence-related Tn5 mutants selected from the 72 shortlisted in the screenings described above (Table 1) were tested in A549 pulmonary cell line. Data obtained so far showed that Tn5 mutants behaved differently in different model systems; thus for the secondary screening, we selected mutants attenuated in at least one *in vitro* phenotypes and in either *C. elegans* or *D. melanogaster* or both. Because all the Tn5 mutants showed a high level of identity in all the *P. aeruginosa* strains analysed *in silico* (Table S2), the level of targets conservation was not used as a selection criterion. As part of the validation of the screening strategy, four mutants not attenuated in *C. elegans* and *D. melanogaster* (*tgpA*, PA3613, PA4265 and PA5156) were also included (Table 1). Thus, a total of 28 Tn5 mutants were tested for cell invasion and cytokine [interleukin (IL)-8] production in the A549 cell line. From the selected Tn5 mutants, 17 showed attenuation in both mammalian cell assays, 7 were attenuated in one of them and 4 in none (Table 1 and Fig. 2). These results suggest that in many cases, the *in vitro* and non-mammalian screenings are not predictive of virulence attenuation in mammalian cell models.

### Quantitative virulence analysis of the selected *P*. *aeruginosa* Tn5 mutants and their impact on a murine model of pneumonia

**Figure 3:**
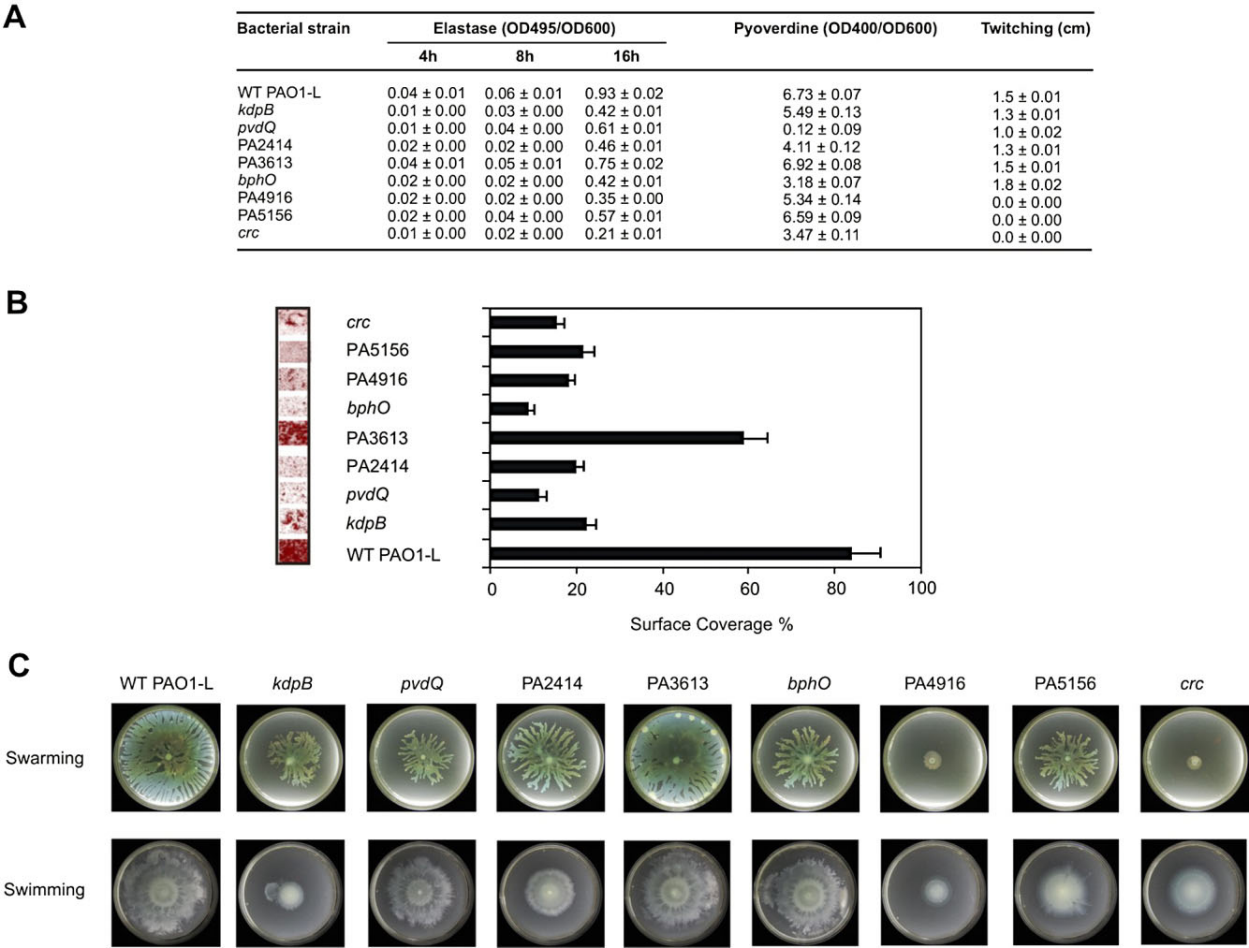
*In vitro* phenotypic characterization of *P*. *aeruginosa* PAO1-L and selected targets. Eight Tn5 mutants (*kdpB*, PA2414, *pvdQ*, PA3613, *bphO*, PA4916, PA5156, *crc*) were selected for further characterization.A. Protease, elastase, pyocyanin and pyoverdine production was determined in supernatant from culture grown to stationary phase using colorimetric assays. For twitching motility, cells were inoculated with a toothpick from an LB agar plate onto a twitching plate (tryptone broth plus 1% agar). The diameter of the twitching zone was determined. Values of triplicate cultures are given.B. Biofilm formation was assessed using a bioflux microfluidic channel. Biofilm cells were continuously grown in 10% LB at 37°C for a period of 14 h. Left panel shows a confocal microscope picture of the biofilms and right panel the representation of the surface coverage for each mutant. Standard deviations are based on the mean values of six images taken in random locations in the microfluidic channel.C. For swarming and swimming motility, cells were inoculated from a 16 h LB culture onto swarming or swimming plates containing 0.5% or 0.3% agar respectively.

As part of the secondary screening, quantitative and more extensive virulence assays were carried out to validate the results obtained so far in *in vitro* and non-mammalian models in a selection of *P. aeruginosa* Tn5 mutants. Eight Tn5 mutants were selected with the following criteria: (i) mutations in genes with known (*kdpB*, *pvdQ*, *bphO*, *crc*) and unknown function (PA3613, PA2414, PA4916, PA5156) and (ii) attenuated in either *C. elegans* (*pvdQ*, PA2414, *bphO*), *D. melanogaster* (PA4916) or both (*kdpB*, *crc*) or not attenuated (PA3613, PA5156). As part of the validation of the screening strategy, two mutants not attenuated in *C. elegans* and *D. melanogaster* were also included, one of which (PA3613) was not attenuated in the mammalian cell models, whereas the other (PA5156) was attenuated (Table 1). These *P. aeruginosa* mutants were subjected to: (i) quantitative *in vitro* analysis for the attenuation of virulence traits including biofilm formation, (ii) determination of *C. elegans* and *D. melanogaster* killing curves, and, finally, (iii) pathogenicity in murine models (Fig. 1A). All the mutants tested showed a reduction in elastase production, especially at 16 h of growth, although at different levels (Fig. 3A). The *crc* mutant showed the lowest elastase levels, whereas the PA3613 mutant produced the most. With regard to pyoverdine, the highest decrease in the production of this siderophore was shown by the *pvdQ* mutant with the PA3613 and PA5156 mutants showing similar levels to the wt PAO1-L. Twitching, swimming and swarming motility was also tested in all mutants in relation to the wild type. The PA3613 mutant showed no reduction in motility as anticipated. In contrast, the PA4916 and PA5332-*crc* mutants showed a drastic reduction in all three forms of motility (Fig. 3A and C). All the mutants tested showed a significant reduction in the ability to form biofilms with the PA3613 once more showing the least reduction (Fig. 3B).

**Figure 4:**
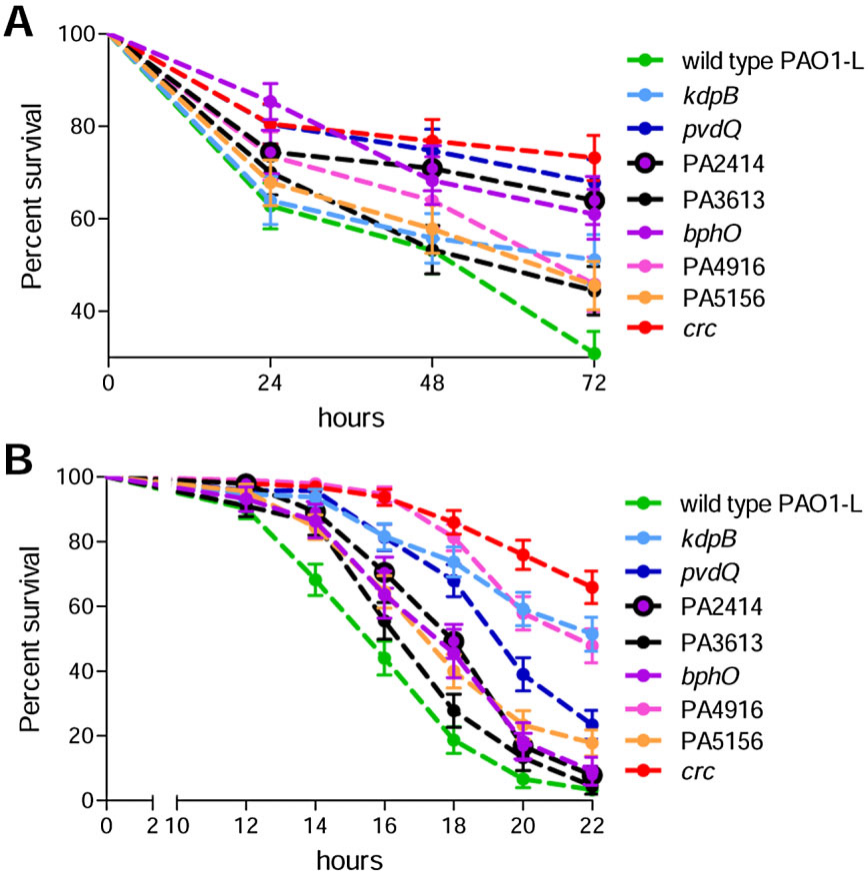
Virulence of *P*. *aeruginosa* PAO1-L and selected targets in *C*. *elegans* and *D*. *melanogaster* disease models. (A) Lethality curves of 72 h in the *C*. *elegans* disease model of eight selected Tn5 mutants (*kdpB*, PA2414, *pvdQ*, PA3613, *bphO*, PA4916, PA5156, *crc*); (B) 22 h lethality curves in *D*. *melanogaster*. The results presented here show the mean values and standard errors calculated from three independent experiments.

The impact of the eight selected *P. aeruginosa* mutations in non-vertebrate models was confirmed following their hosts survival rates over a period of 72 h for *C. elegans* (Fig. 4A) and 22 h for *D. melanogaster* (Fig. 4B). The killing curves confirmed the results shown in Table 1. Interestingly, three mutants, *pvdQ*, PA2414 and *bphO*, were strongly attenuated in the *C. elegans* model, but only weakly or not at all in the *D. melanogaster* model. The *crc* mutant was found to be the most attenuated strain in both infection models.

**Figure 5:**
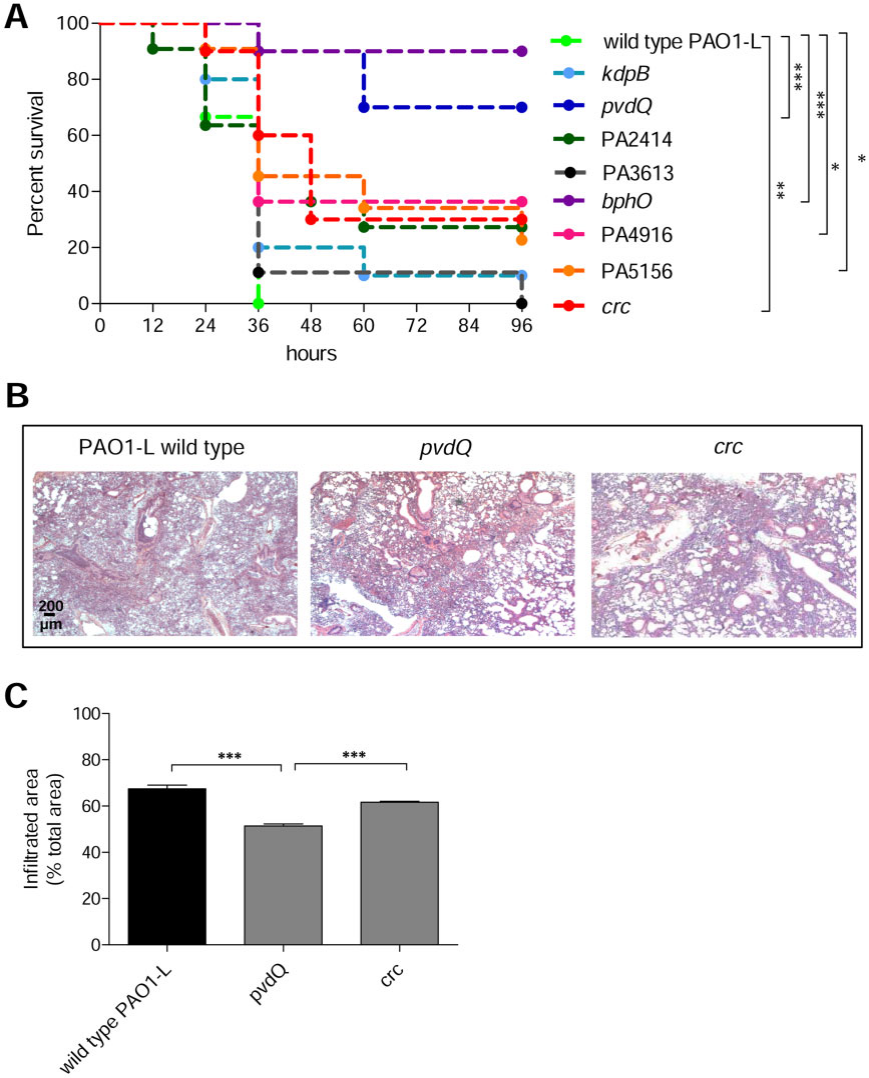
Virulence of *P*. *aeruginosa* PAO1-L and selected targets in a mouse model of acute lung infection in C57BL/6NCrl mice. C57BL6/NCrl 8 weeks old purchased from Charles River Laboratory were intratracheally injected with 5 × 10^6^ CFU of PAO1-L wild type or mutants.A. Survival was monitored up to 96 h. Two independent experiments were pooled. Statistical analysis was calculated for pair wise comparisons between wild-type and mutant strains. *P < 0.05, **P < 0.01, ***P < 0.001, Mantel–Cox test.B. Lung histopathology was performed after 24 h from infection for wild-type PAO1-L, *pvdQ* mutant and *crc* mutant.C. Quantification of infiltrated areas as a percentage of total tissue area with mean ± SEM is shown. Statistical analysis was calculated for pair wise comparisons between wild-type and mutant strains. ***P < 0.001, Mann–Whitney.

The final validation of the attenuation observed in the *in vitro* and non-mammalian hosts virulence assays in the eight mutants selected (*kdpB*, *pvdQ*, *bphO*, *crc*, PA2414, PA4916, PA5156, PA3613) was carried out by assaying their levels of lethality in a murine model of acute lung infection (Fig. 5). After having assessed the first lethal dose [5 × 10^6^ colony-forming units (CFU)] for PAO1-L in C57BL/6NCrlBR mice (Fig. S2), the 8 Tn5 mutants were compared (Fig. 5). PAO1-L was fully lethal within 36 h, whereas the lethality of all the mutants tested was significantly lower and temporally shifted, with the exception of the PA3613 mutant (Fig. 5A). Based on survival curves, *pvdQ*, *bphO*, PA4916, PA5156, *crc* were significantly less lethal in mice when compared with PAO1-L. Histopathological analysis showed that the wild-type PAO1-L strain induced higher inflammation and more severe lesions in comparison with the *crc* and *pvdQ* mutants (Fig. 5B). The area infiltrated by inflammatory cells by quantitative analysis was significantly higher in the lungs infected with the wild-type PAO1-L than with the mutant *pvdQ* (Fig. 5C).

**Figure 6:**
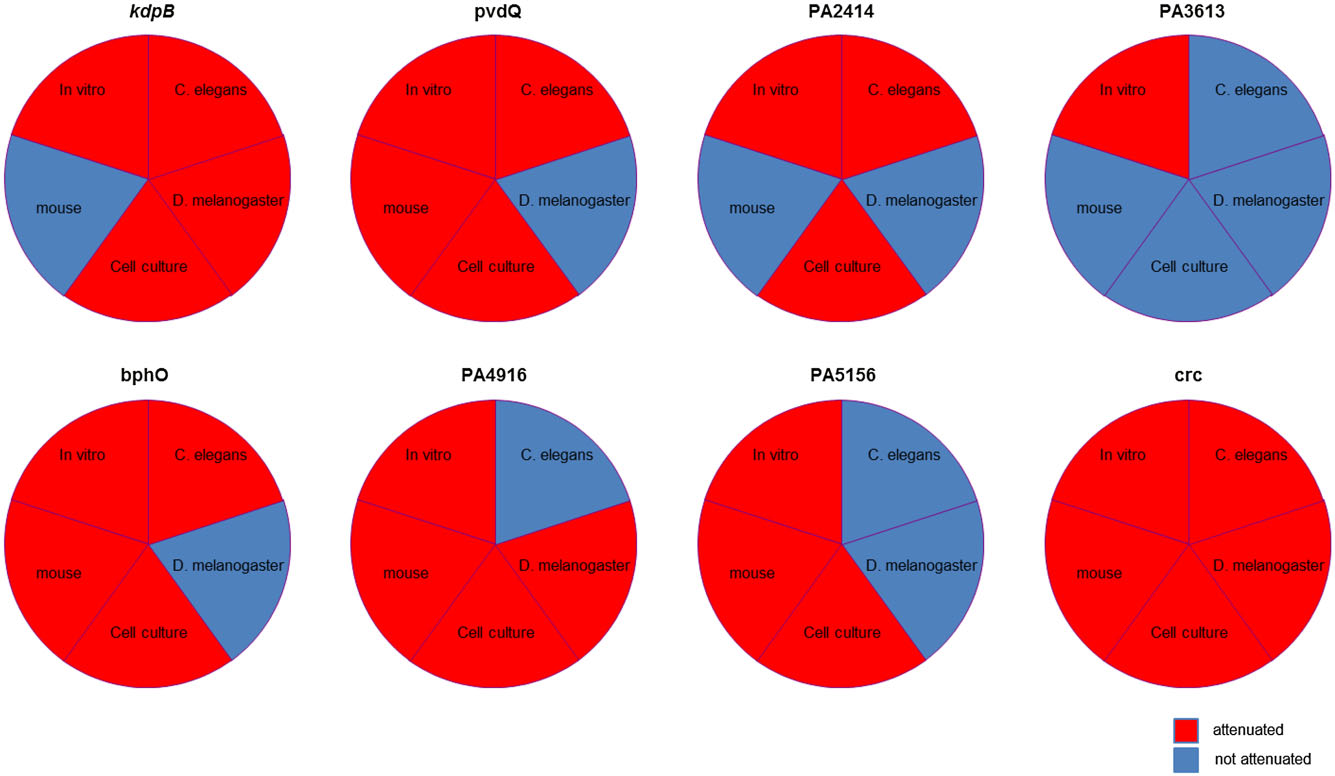
Poor correlation of *P*. *aeruginosa* virulence between different model systems. Summary of the results obtained *in vitro*, *C*. *elegans*, *D*. *melanogaster*, cell culture and mouse. Red indicates attenuation in virulence (at least one assay for in vitro and cell culture); blue indicates non-attenuation.

Overall, our data indicated that virulence determinants are host specific as evidenced by the summary of the results obtained in different model systems in Fig. 6.

## Discussion

This study set out to identify novel virulence genes in *P. aeruginosa* using a multi-host screening strategy with the ultimate goal of selecting for novel anti-virulence targets. This strategy consisted on the initial screening of a Tn5 library, constructed within the current study, using a set of *in vitro* virulence assays to select mutants with pleiotropic effects. The selected mutants were subsequently tested in a sequential fashion on non-mammalian (e.g. *D. melanogaster* and *C. elegans*) and mammalian (e.g. airways cell lines and mice) disease model systems. Considering that *P. aeruginosa* has a variety of clinical presentations and shows an extraordinarily broad host range, different infection models would enable better understanding of which genes are major contributors to virulence and whether these genes are host dependent or not. We considered this question worthy of investigation because it seemed likely to us that the virulence factors of an opportunistic multi-host pathogen might have a superior clinical relevance when compared with the virulence factors of host-specific pathogens.

Previous studies on the identification of *P. aeruginosa* virulence genes *in vivo* using different disease models have provided important information on pathogenicity but were limited to either the number of mutants tested or the use of a single host. Genome-wide screening have been carried out mainly in *C. elegans* (Feinbaum *et al*., 2012), *D. melanogaster* (Kim *et al*., 2008), *Dictyostelium* amoeba (Alibaud *et al*., 2008), mice (Bianconi *et al*., 2011) and rats (Potvin *et al*., 2003) (Winstanley *et al*., 2009). Although in the study with *Dictyostelium* they used a range of hosts including this amoeba, *D. melanogaster* and mice, the study only targeted a small proportion of the genome as it was limited to a redundant mini-Tn5 mutant library of 2500 clones in a *lasR* mutant clinical isolate, all of which were tested in the amoeba model, but only 4 in *D. melanogaster* and mice. In contrast the screening presented in this manuscript was much more extensive, using a total of 57 360 Tn5 redundant mutants, representing an estimated 95% of the genome (Liberati *et al*., 2006) with a total of 404 independent pleiotropic mutants found attenuated in two or more virulence traits. It is well known that *P. aeruginosa* isolates from different environments and clinical settings show a heterogeneous *in vitro* production of various known virulence factors. However, some *P. aeruginosa* virulence-associated factors, like swarming motility, protease and pyocyanin production have been found in most isolates (Tielen *et al*., 2011; Mayer-Hamblett *et al*., 2014) and hence were selected in this study for the *in vitro* screening, as they are also of major relevance to infection. The pleiotropic mutants were then tested for their ability to kill *D. melanogaster* and *C. elegans* and a set of 108 attenuated mutants in putative virulence-related mutants (corresponding to 68 genes) identified. Mapping of the Tn5 insertion sites in each of the mutants identified revealed multiple strains with the mutation in the same gene, indicating that the screen was saturating. The 68 virulence-related genes found are broadly distributed across 16 functional classes and conserved within *P. aeruginosa* strains of different origin and in their majority across the Gram-negative pathogens *E. coli* and *B. cenocepacia*. Furthermore, practically all these virulence-related genes did not have homologues in humans, which increases the chances of the future identification of inhibitors with selected toxicity for their gene products. The majority of the virulence-related genes identified belonged to the functional category of hypothetical, unknown and unclassified genes (24 genes, 35.8% of the total), suggesting that there is still a large proportion of unknown genes remaining to be discovered within the unexplored part of the *P. aeruginosa* genome. Our screening also identified a number of mutants with insertions in genes already shown to be required for virulence in *P. aeruginosa* supporting the robustness of the screening method used in this study. Some of these genes are known to be involved in antibiotic resistance, biofilm formation, motility, virulence factors production and secretion, metabolism and quorum sensing regulation. Only 10 genes already found in previous genome-wide screens were identified in the present study. This limited overlap supports the idea of a bias toward the disease model used. Of notice, the overlaps found were only with screens carried out in *C. elegans* (Feinbaum *et al*., 2012), mice (Bianconi *et al*., 2011) or rats (Potvin *et al*., 2003; Winstanley *et al*., 2009); no overlap with previous screens using *D. melanogaster* (Kim *et al*., 2008) were identified, possibly due to the differences between the *P. aeruginosa* strains used in these studies. Nevertheless, as mentioned before, the screening method presented in this manuscript favours the identification of mutants with attenuated virulence in multi-host systems rather than in a single host.

Similar to previous screens, in this study, a number of genes involved in adaptation to environmental stress were also identified. Those include a large number of metabolic genes involved in the generation and transport of precursor metabolites and energy (*fdhE*, *ptsP*, *xdhB*, *bphO*, *nqrB*, *nagE* and PA2414), and genes involved in DNA synthesis and modification (*smpB*, *pdxA*, PA3867, PA3950 and PA4282). Interestingly, genes playing a part in amino acid synthesis and metabolism were also identified (*pauB1* coding for a D-amino acid oxidase, *hisD* coding for a histidinol dehydrogenase, PA3139 coding for a threonine-phosphate-decarboxylase). This could maximize the utilization of the amino acids present in different environments such as those encountered in the CF lung (Thomas *et al*., 2000). The identification of a number of mutants in genes involved in biofilm formation (*psl*, *mucP* and *pelG*), type IV and VI secretion systems (*xcpT*, *pilD* and *tssA1*) and stress response (*phoR*, *rpoS* and *parR*) also illustrate their importance for survival of *P. aeruginosa* in the ‘host environment’.

It has recently been argued that to understand the bacterial infection process, it is key to consider the metabolic transactions taking place and not simply ignore the metabolic genes identified in screenings searching for virulence factors (de Lorenzo, 2015). These metabolic processes vary from host to host and our manuscript clearly supports this argument, because mutants in certain metabolic genes show disease attenuation only in specific hosts. Hence, the nutrients provided by different environments are going to be key determinants in the infection process.

Surprisingly, this study revealed that a set of above mentioned *P. aeruginosa* virulence factors are not involved in pathogenicity in multiple hosts. The most striking difference was detected between the two non-mammalian models used in our study where the majority of virulence genes attenuated in *C. elegans* were not in *D. melanogaster* (43 out of 72 genes, about 60%). Furthermore, other discrepancies were found when comparing virulence attenuation between A549 cells, *C. elegans* and *D. melanogaster*. These results are quite surprising because it has been described that there is conservation between innate immune pathways in *D. melanogaster*, *C. elegans* and mammals (Ferrandon *et al*., 2007; Engelmann and Pujol, 2010).

Finally, while testing selected mutants in murine pneumonia models, the current study found agreement between the presence or absence of attenuation for some targets between *C. elegans* and mice (*pvdQ*, *bphO*, *crc*, PA3613), whereas others showed different results in the two models. PA2414 was attenuated in *C. elegans* but not in mice, and PA4916 and PA5156 were attenuated in the mouse model of acute infection but not in *C. elegans*. Similarly, agreement was observed between the presence or absence of attenuation for some targets in *D. melanogaster* and mice (*kdpB*, *pvdQ*, PA2414, PA3613, PA4916 and *crc*), whereas others showed different results (*bphO* and PA5156). *BphO* was attenuated in *C. elegans*, but not in mice, and PA5156 was attenuated in the mouse model of acute infection, but not in *C. elegans*. The data presented in this manuscript show that virulence genes, which were identified *in vitro* or in non-mammalian infection models, may not be relevant for pathogenesis in mammalians (Fig. 6). Our results complement recent findings by Hilker *et al*., (2015) in which they showed that *P. aeruginosa* strains isolated from human infections and the environment evoked a significantly different virulence gradient in the lettuce, *Galleria mellonella* and mice models. Interestingly they found that some strains that had never been in contact with a human host were more virulent in the disease models they used than some of those isolated from human. Furthermore, in line with the results of our manuscript, the degree of virulence between the *P. aeruginosa* strains tested varied enormously depending on the disease model used.

In the context of infections caused by *P. aeruginosa*, virulence is mediated by a wide network of regulators that sense the environment and the physiological state of the cell and adjust the transcription of specific genes resulting in significant phenotypic changes. The use of non-mammalian infection models has several downsides when compared with the mammalian host. These include the differences in incubation temperatures and the lack of specific target organs, key receptors and pathways, which can not only alter the behaviour of the bacterial pathogen, but also affect the knowledge extrapolation into the human host. However, although rodents are the first choice for understanding infectious diseases in humans, screening a large amount of targets in mouse models is not feasible. Despite the downsides described above, non-mammalian models remain a useful surrogate hosts for initial large-scale screening, but our results indicated that final validation should be carried out always in mammalian hosts. Thus, a multifaceted bottleneck approach, by using a sequential cascade of model systems, may be essential for the selection of anti-virulence drug targets. Whether the virulence targets identified in this study will have a clinical relevance remains to be fully established. The approach employed in this work, although representing a step forward toward the understanding of *P. aeruginosa* pathogenesis, does not totally reflect the complex scenarios taking place in infected patients. It has been recognized that genes required for pathogenicity in one strain of *P. aeruginosa* may neither be required for nor predictive of virulence in other strains (Lee *et al*., 2006). In addition, several lines of evidence are transforming the view of infectious diseases from strictly pathogen-centric to the one incorporating the host environmental and the genetic determinants that modulate immune responses (Chapman and Hill, 2012). Thus, a wide combination of *P. aeruginosa* strains from different origins and validation in various disease models including mammalians are ideally required when searching for novel virulence targets to gain a better understanding of the possible impact they may have in the human host.

### Conclusions

Overall, our data provide an important message to be taken into consideration when searching for novel anti-virulence targets to develop new treatments against *P. aeruginosa* infections. *P. aeruginosa* virulence is a highly complex, multifactorial process requiring the coordinated activity of many bacterial gene products that production and activity can significantly vary depending on the infection model used and hence the host environment. The idea of a core set of virulence factors common to all infection models may only apply to a very limited number of them. The spectrum of virulence factors that play a role in a given host model depends on a wide range of factors including the intrinsic characteristics of the site of infection, the type of the immune response and the phase of infection. Many virulence factors from *P. aeruginosa* are likely to play very distinct roles according to the sites of infection, posing an even greater challenge for the development of novel personalized therapeutics.

## Experimental procedures

### Ethics statement

Mice studies were conducted according to protocols approved by the San Raffaele Scientific Institute (Milan, Italy) Institutional Animal Care and Use Committee (IACUC) and adhered strictly to the Italian Ministry of Health guidelines for the use and care of experimental animals.

### Bacterial strains, growth conditions, plasmids and general DNA manipulation

The bacterial strains and plasmids used are listed in Table S1. *Escherichia coli* and *P. aeruginosa* were routinely cultured in Luria–Bertani (LB) at 37°C under vigorous shaking (200 r.p.m.). Media were solidified with 1.8% agar. The antibiotics were added, when required, to final concentrations of 20 μg ml^−1^ for gentamicin, 15 μg ml^−1^ for nalidixic acid and 20 μg ml^−1^ for streptomycin. Tetracycline was added into media at the final concentrations of 125 μg ml^−1^ for *P. aeruginosa* and 10 μg ml^−1^ for *E. coli*.

Genomic DNA isolation was performed using a Wizard Genomic DNA purification kit (Promega). Plasmid DNA isolation was performed using a Qiagen Midi Kit. All other DNA manipulation techniques including analysis, digestion, ligation and introduction of DNA into host cells by electroporation were performed as described by Sambrook and Russel (2001).

### Transposon mutagenesis

Transposon mutants were generated by mating *P. aeruginosa* PAO1 obtained from D. Haas (Department of Microbiology, University of Lausanne, Switzerland) (referred to as PAO1-L) (Heurlier *et al*., 2003) with *E. coli* strain λ*pir* S17.1 carrying pLM1 derived from pRL27 (Larsen *et al*., 2002). *Pseudomonas aeruginosa* mutants were selected by plating on LB agar containing gentamycin (20 μg ml^−1^), streptomycin (500 μg ml^−1^) and nalidixic acid (15 μg ml^−1^). After incubation at 37°C for 16 h mutant colonies were picked using a Flexis (Genomics Solutions) colony-picking robot. Colonies were arrayed into 94-well plates containing 200 μl of 15% glycerol in LB. The microtitre plates were sealed using a gas-permeable membrane (Corning), incubated at 37°C for 16 h and then stored at −80°C.

### Mutant selection

The *P. aeruginosa* mutant strains were screened for alterations in pyocyanin production, swarming motility and/or alkaline protease activity by visual examination with the aid of the colony picker described in the previous section, which was programmed to replicate the bacterial cultures from 96-well plates into Omni Trays (Fisher Scientific) containing various solidified media: LB to assess pyocyanin production through changes in blue coloration, swarming agar (Rampioni *et al*., 2010), or LB containing 5% (w/v) of skimmed milk for determination of protease activity.

### Quantitative assays for phenotypic alteration

Pyocyanin and pyoverdine production, protease and elastase activity, motility and biofilm formation were evaluated as described (Essar *et al*., 1990). Details are reported in the Supporting information.

### Transposon insertion location

To determine the transposon insertion point in the chromosome of the selected Tn5 mutants, total genomic DNA was isolated and digested with *EcoR*I, which nucleotide sequence is absent within pLM1. Digested genomic DNA fragments were re-circularized and, upon electroporation in *E. coli* λ*pir* S17.1, selected for gentamicin resistance. DNA insert regions flanking the Tn5 were sequenced using primers tpnRL17-1 (5′-AACAAGCCAGGGATGTAACG-3′) and tpnRL17-2 (5′-CAGCAACACCTTCTTCACGA-3′), at the Biopolymer Synthesis and Analysis Department, Queens Medical Centre, Nottingham, UK. DNA sequence analysis was performed using software packages provided by the National Centre for Biotechnology Information BLAST network server.

### *C.* *elegans* and *D*. *melanogaster* virulence assays

Nematode slow-killing assays were performed essentially as previously described (Lorè *et al*., 2012). The *D. melanogaster* disease model was used as previously described (Apidianakis and Rahme, 2009). Details are reported in the Supplementary information.

### *In silico* analysis

Comparative protein sequence analysis was carried out against the six sequenced genomes (PA14, LESB58, PA7, 2192, C3719 and PACS2) of *P. aeruginosa* and against *E. coli* K12 and *B. cenocepacia* J2315 in order to check for the presence and conservation of virulence genes in other *P. aeruginosa* genomes and in other bacterial species. The blastp program from NCBI (http://blast.ncbi.nlm.nih.gov/Blast.cgi?PAGE=Proteins) was used for protein homology analysis by aligning the amino acid sequence of PAO1 against other genomes. Furthermore, genes identified in our screen were then checked for their presence in the annotated Virulence Factor Database (http://www.mgc.ac.cn/VFs/) (Chen *et al*., 2005).

### Cell culture, invasion assay and IL-8 secretion

The A549 (human type II pneumocytes) cell line was purchased from ATCC CCL-185 and cultured as described (Pirone *et al*., 2008). Bacteria invasion assay was performed using Polymvxins B (100 μg ml^−1^) (Sigma) protection assay with minor modifications (Bianconi *et al*., 2011) and as detailed in the Supporting information. IL-8 secretion was performed as reported previously (Bianconi *et al*., 2011).

### Mouse model of acute *P*. *aeruginosa* infection

C57Bl/6NCrlBR male mice (20–22 g) were purchased from Charles River Laboratories, Italy. Acute *P. aeruginosa* infection was performed as previously described (Lorè *et al*., 2012) and detailed in the Supporting information. Histopathology was performed according to standard procedures.

### Statistical analysis

Results are presented as mean ± standard error of the mean (SEM). Statistical calculations and tests were performed using Student’s paired *t*-test, and Log rank Mantel–Cox test in order to determine the significance of differences in means between pairs.

## Supporting Information

**Fig. S1.** Enrichment of functional categories in primary screen. **Fig. S2.** Dose dependent survival curve in C57Bl/6 mice infected with PAO1-L. **Table S1.** Bacterial strains and plasmids used in this study. **Table S2.** *In silico* comparisons between *P. aeruginosa* strains, other gram-negative bacteria, and humans.
