# Supplementary figures and images for: Integrated whole-genome screening for Pseudomonas aeruginosa virulence genes using multiple disease models reveals that pathogenicity is host specific
Source: Environ Microbiol. 2015 May 14;17(11):4379–93. doi: 10.1111/1462-2920.12863 (PMC4676916; doi:10.1111/1462-2920.12863)

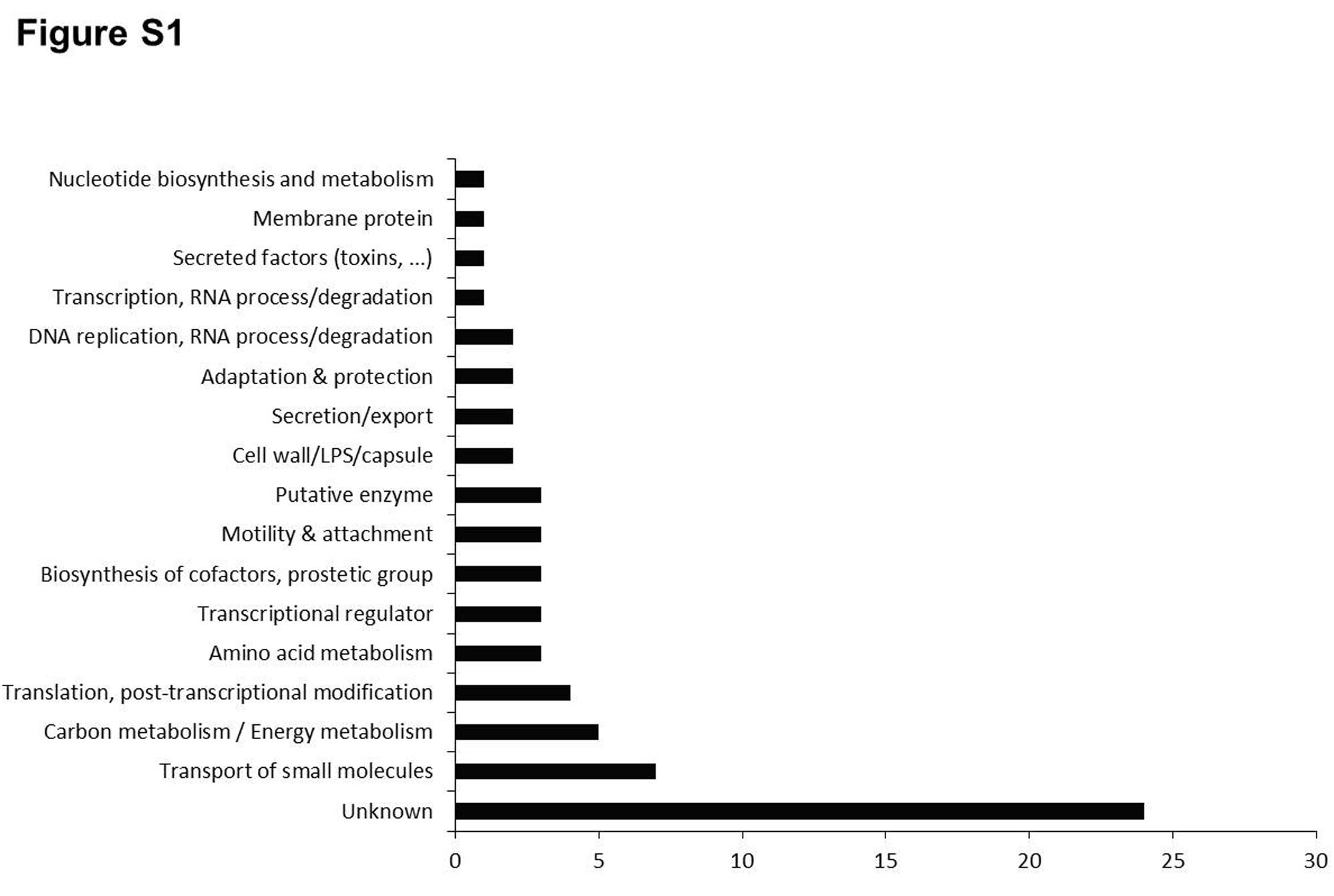

Supplement: Supplementary file 1 — Fig. S1. Enrichment of functional categories in primary screen. Fig. S2. Dose dependent survival curve in C57Bl/6 mice infected with PAO1-L. Table S1. Bacterial strains and plasmids used in this study. Table S2. In silico comparisons between P. aeruginosa strains, other gram-negative bacteria, and humans. [file emi0017-4379-sd1.zip › EMI_12863_Supp-0002-Figure-S1.jpg]

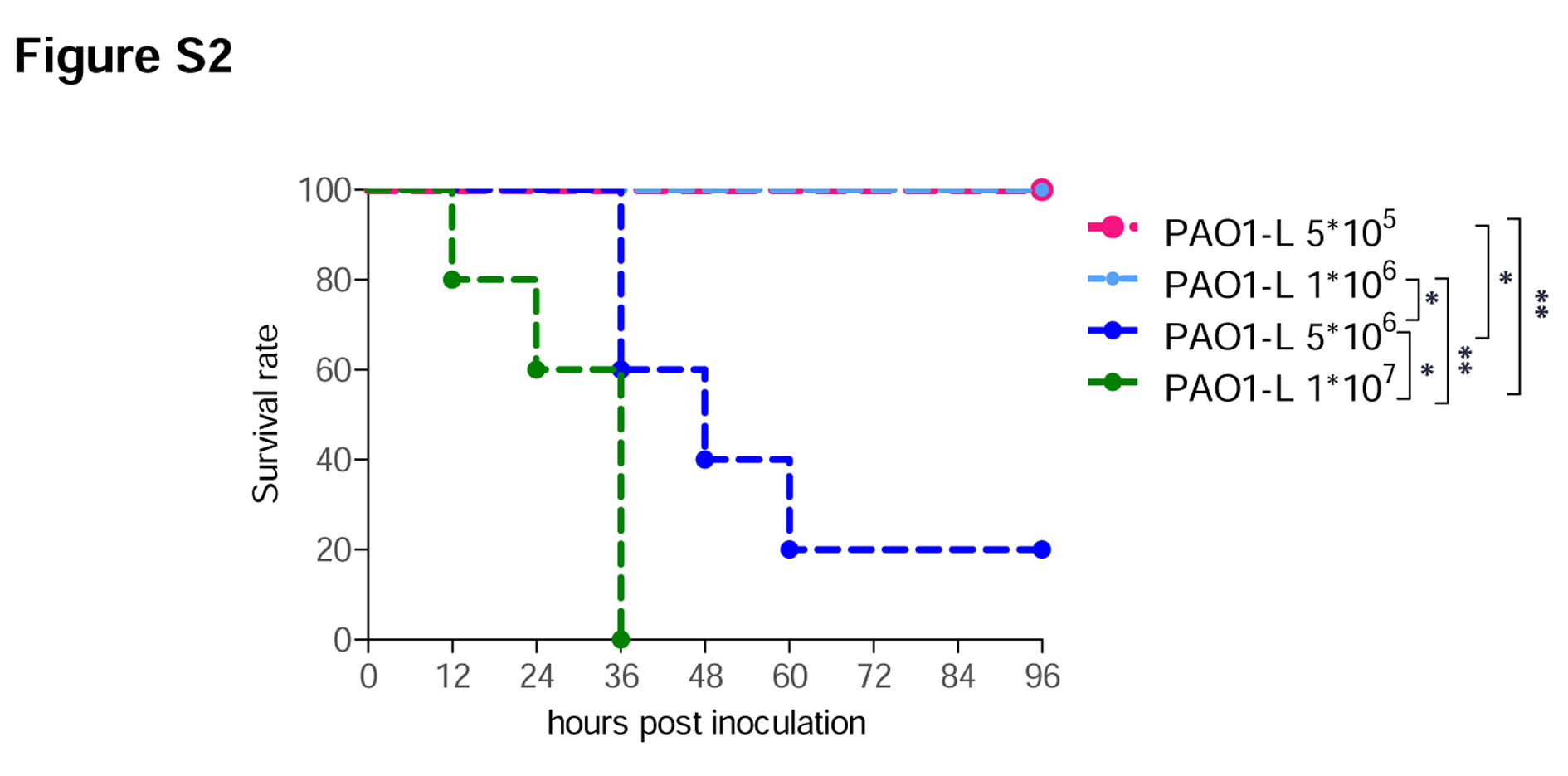

Supplement: Supplementary file 1 — Fig. S1. Enrichment of functional categories in primary screen. Fig. S2. Dose dependent survival curve in C57Bl/6 mice infected with PAO1-L. Table S1. Bacterial strains and plasmids used in this study. Table S2. In silico comparisons between P. aeruginosa strains, other gram-negative bacteria, and humans. [file emi0017-4379-sd1.zip › EMI_12863_Supp-0003-Figure-S2.jpg]
