# Supplementary material for: Integrated whole-genome screening for Pseudomonas aeruginosa virulence genes using multiple disease models reveals that pathogenicity is host specific
Source: Environ Microbiol. 2015 May 14;17(11):4379–93. doi: 10.1111/1462-2920.12863 (PMC4676916; doi:10.1111/1462-2920.12863)
Supplement: Supplementary file 1 — Fig. S1. Enrichment of functional categories in primary screen. Fig. S2. Dose dependent survival curve in C57Bl/6 mice infected with PAO1-L. Table S1. Bacterial strains and plasmids used in this study. Table S2. In silico comparisons between P. aeruginosa strains, other gram-negative bacteria, and humans. [file emi0017-4379-sd1.zip › EMI_12863_Supp-0001-Tn5_09S_EnMicro.doc]

**Integrated whole genome screening for *Pseudomonas aeruginosa* virulence genes using multiple disease models reveals that pathogenicity is host specific**

Jean-Frédéric Dubern^1¥^, Cristina Cigana^2¥^, Maura De Simone^2^, James Lazenby^1^, Mario Juhas^3^, Stephan Schwager^3^, Irene Bianconi^2^, Gerd Döring^4^**^$^**, Leo Eberl^3^, Paul Williams^1^, Alessandra Bragonzi^2¥*^ and Miguel Cámara^1¥*^

**Supporting Information**

**Experimental Procedures**

***Phenotypic characterization of Tn-5 mutants*.**

***Pyocyanin and pyoverdine production.*** In order to test pyocyanin production, *P. aeruginosa* strains were grown in LB at 37^o^C for 16h. The concentrations of pyocyanin were determined in 5 ml culture supernatants as described previously (Essa*r et a*l., 1990). For pyoverdine production, *P. aeruginosa* cells were grown in CAA medium for 24h (Ongen*a et a*l., 2002 ). Pyoverdine production was assessed by determination of the optical density of culture supernatants at 400 nm.

***Protease and elastase activity.*** Protease and elastase activities were assessed using a colorimetric method (Ohma*n et a*l., 1980). Briefly, 100 µl of overnight cultures were added to a buffer containing 100 mM Tris, 1 mM CaCl_2_ (pH 7.5) and 5 mg. ml^-1^ of the substrates azocasein (Sigma) for protease detection assay or elastin congo red (Sigma) for elastase detection assay. The enzymatic reaction mixtures were incubated at 37^o^C, and stopped after 15 min using 500 μl of trichloroacetic acid 10% (v/v) for protease and after 2 h using 100 μl of 120 mM EDTA for elastase. The level of enzymatic activity was determined by reading the optical density of clear supernatant at 400 mM for protease and 495 mM for elastase.

***Motility assays.***

***Twitching.*** Twitching assays were done using Difco LB broth solidified with 1% (w/v) Difco agar. Twitching plates were briefly dried and strains were stab inoculated with a sharp toothpick to the bottom of the Petri dish from an overnight-grown LB agar (1.5%, w/v) plate. After incubation at 37°C for 24 h, the zone of motility at the agar/Petri dish interface was measured.

***Swarming.***

For swarming motility assay, a volume of 5 µl of an overnight (16 h) bacterial culture was spotted on swarming agar plate containing 5 g. l^-1^ Bacto agar (Difco), 8 g. l^-1^ Nutrient broth N^o^2 (Oxoid), and 0.5% (w/v) glucose (Sigma), and incubated at 37^o^C for 16 h.

***Swimming.***

The media used for swimming assay was nutrient broth N^o^2 (Oxoid), 0.5% (w/v) glucose (Sigma) and 0.3% (w/v) agarose (GIBCO/BRL). Swimming plates were inoculated with bacteria from an overnight culture in LB agar (1.5%,w/v) plates at 37°C with a sterile toothpick. The plates were then sealed with parafilm (3M) to prevent dehydration and incubated at 25°C for 12h.

***Biofilm formation.***

The ability of *P. aeruginosa* PAO1-L to form biofilm was analysed using the Bioflux system (Fluxion). Biofilms were grown under continuous flow in microfluidic channels. *P. aeruginosa* PAO1-L wild type and its transposon mutants derivatives tagged with Tn7-*egfp* were grown at 37^o^C for 16h in 2 ml Müller Hinton (MH) (Oxoid) broth. The culture was diluted 1/1000, grown for a further 4h before setting up the biofilm experiment, and diluted to OD 0.05 in 7% MH broth. The biofilm was allowed to form in 10% MH broth at a flow rate of 63μl/h (corresponding to a share rate of 2 dyn/cm^2^) at 37^o^C for 15h. The microfluidic channel was assayed under a Laser Scanning fluorescent Microscope (LSM2, Zeiss). Biofilms were visualised using light microscope and *egfp* mode at an excitation wavelength of 488nm. Imaging was carried out using Zen 2009 imaging software (Zeiss). A total of 5 replicate images were taken randomly along the microfluidic channel with a Z-stack of 75 images. Biofilm images were analysed for biomass using Comstat 2 statistical software (Heydorn, 2000). An automatic thresholding was applied using the Ortsus’s method.

***C. elegans* virulence assays.** Briefly, worms of the *C. elegans* strain DH26 were synchronized into L4 larval stadium by egg preparation, which was followed by incubation of isolated eggs on *E. coli* OP50 feeding plates at 20°C for around 76 hours. Subsequently, L4 larvae were transferred onto lawns of the *P. aeruginosa* mutant strains grown in the 6-well plates (approximately 30 worms per well) and incubated at 25°C. The surviving worms were counted after 24, 48 and 72 hours with the aid of a Stereolupe Stemi SV 6 (Zeiss). The pathogenicity of the *P. aeruginosa* mutants was determined from the survival rates of *C. elegans* in three independent replicates.

***D. melanogaster* virulence assays***. D. melanogaster* Canton-S wild type flies were bred on standard cornmeal sucrose medium at room temperature. Male and female flies (2-9 days old) were used as infection hosts. Bacterial strains were grown in LB to an OD_600_ of 1.0. The cells were harvest, washed and adjusted with 10 mM MgSO_4_ to an OD_600_ of 2.0. Flies were anaesthetized with ether and bacterial suspensions were inoculated into the thorax using a tungsten needle. For each trial, triplicate sets of 15 flies were used and the experiment was performed in duplicate, except when no attenuation was found. The negative control flies were inoculated with a 10 mM MgSO_4_ solution. If more than 5 flies died because of injury caused during pricking, the experiment was repeated. The pricked flies were incubated at 26°C and counted every hour between 11h to 22h post infection.

**Cell culture, invasion assay and IL-8 secretion.** *P*. *aeruginosa* strains, grown to mid-exponential phase, were used to infect cell monolayers at a 100:1 multiplicity of infection for 2h. The monolayers were washed with PBS, treated with antibiotic for 2h, washed, lysed with H_2_0 and plated on TSB-agar plates (Difco). IL-8 levels were determined in supernatants collected from the cell cultures described above using an ELISA kit (Biosource Europe). According to the manufacturer the sensitivity of the assay is less than 0,7 pg/ml. Values were normalized to 10^6^ cells and results expressed as mean +/- SD.

**Mouse model of acute *P. aeruginosa* infection.** Mice were housed in filtered cages under specific-pathogen conditions and permitted unlimited access to food and water. Prior to animal experiments, the *P. aeruginosa* mutant strains were grown for 3h to mid exponential growth phase. Bacteria were then harvested, washed twice with sterile PBS and the OD of the bacterial suspension adjusted to an OD 600 nm. Planktonic bacteria were resuspended in sterile PBS to the desired dose for infection of 5x10^6^ CFU/mouse. Mice were anesthetized and the trachea directly visualised by a ventral midline incision, exposed and intubated with a sterile, flexible 22-gr cannula attached to a 1 ml syringe accordingly to established procedures (Lor*è et a*l., 2012). A 60 µl inoculum of 5x10^6^ CFU were implanted via the cannula into the lung, with both lobes inoculated. After infection, survival was monitored twice a day for up to four days. Mice that lost >20% body weight and had evidence of severe clinical disease, such as scruffy coat, inactivity, loss of appetite, poor ambulation, dehydration or painful posture, were sacrificed by CO_2_ administration before termination of the experiment. In another group of mice, the lungs were excised and processed for histopathology.

**Histological analysis.** Mice were sacrificed by CO_2_ administration after 24h of infection, lungs were removed en bloc and fixed in 10% buffered formalin at 4°C for at least 24h and processed for paraffin embedding. Longitudinal sections of 5 μm from the proximal, medial and distal lung regions were obtained at regular intervals using a microtome. Sections were stained with haematoxylin and eosin according to standard procedures. Areas of inflammatory cell infiltration and tissue preservation (normal histology) were quantified using Image J software (National Institutes of Health) and reported as a percentage of total area.

**References**

Essar, D., Eberly L, Hadero A, Crawford IP (1990) Identification and characterization of genes for a second anthranilate synthase in Pseudomonas aeruginosa: interchangeability of the two anthranilate synthases and evolutionary implications. . *J Bacteriol* **172**: 884-900.

Heurlier, K., Denervaud V, Pessi G, Reimmann C, Haas D. (2003) Negative control of quorum sensing by RpoN (sigma54) in Pseudomonas aeruginosa PAO1. *J Bacteriol* **185**: 2227-2235.

Heydorn, A., Nielsen AT, Hentzer M, Sternberg C, Givskov M, Ersbøll BK, Molin S. (2000) Quantification of biofilm structures by the novel computer program COMSTAT. *Microbiology* **146**: 2395-2407.

Larsen, R.A., Wilson, M.M., Guss, A.M., and Metcalf, W.W. (2002) Genetic analysis of pigment biosynthesis in Xanthobacter autotrophicus Py2 using a new, highly efficient transposon mutagenesis system that is functional in a wide variety of bacteria. *Archives of Microbiology* **178**: 193-201.

Lorè, N., Cigana C, De Fino I, Riva C, Juhas M, Schwager S, Eberl L, Bragonzi A (2012) Cystic Fibrosis-Niche Adaptation of Pseudomonas aeruginosa Reduces Virulence in Multiple Infection Hosts. *PlosOne* **7**: e35648.

Metcalf, W., Jiang W, Wanner BL. (1994) Use of the rep technique for allele replacement to construct new Escherichia coli hosts for maintenance of R6K gamma origin plasmids at different copy numbers. *Gene* **28**: 1-7.

Ohman, D., Burns RP, Iglewski BH. (1980) Corneal infections in mice with toxin A and elastase mutants of Pseudomonas aeruginosa. *J Infect Dis.* **142**: 547-555.

Ongena, M., Jacques P, Delfosse P, Thonart P (2002 ) Unusual traits of the pyoverdin-mediated iron acquisition system in Pseudomonas putida strain BTP1. *Biometals* **15**: 1-13.

**Figure Legends**

**Figure S1. Enrichment of functional categories in primary screen.** The number of genes in each category is shown based on their PseudoCAP function classes: hypothetical, unknown, unclassified proteins, transport of small molecules, carbon metabolism and energy metabolism, translation and post-transcriptional modification, amino acid metabolism, transcriptional regulators, biosynthesis of co-factors and prosthetic group, motility and attachment, putative enzyme, cell wall/LPS/capsule, secretion and export, adaptation and protection, DNA replication, transcription and RNA process/degradation, secreted factors, membrane protein, and nucleotide biosynthesis and metabolism.

**Figure S2. Dose dependent survival curve in C57Bl/6 mice infected with PAO1-L.** C57BL6/NCrl eight weeks old purchased from Charles River Laboratory were intratracheally injected with 5x10^5^, 1x10^6^, 5x10^6^, 1x10^7^ CFU of PAO1-L wild type. Survival was monitored up to 96 hours. Statistical analysis was calculated for pair wise comparisons between different PAO1-L doses. *p<0.05, **p<0.01, Mantel-Cox test.

**Table**

**Table S1**. Bacterial strains and plasmids used in this study.

| **Strain or plasmid** | **Relevant characteristics** | **Source or reference** |
| --- | --- | --- |
| **Strains** |  |  |
| *P. aeruginosa* |  |  |
| PAO1-L | PAO1 Lausanne collection wild type | (Heurlie*r et a*l., 2003) |
| *E. coli* |  |  |
| λ*pir* S17.1 | Thi proA hsdR recA RP4-2-tet::Mu-1 kan::Tn7integrant (Tp^r^ Sm^r^) | (Metcal*f et a*l., 1994) |
| **Plasmids** |  |  |
| pRL27 | Plasmid harbouring Tn5 transposon, Km^r^ | (Larse*n et a*l., 2002) |
| pLM1 | Plasmid harbouring Tn5 transposon derived from pRL27, Gent^r^ | (Larse*n et a*l., 2002) |

# Table S2. *In silico* comparisons between *P. aeruginosa* strains, other gram-negative bacteria, and humans. Predicted amino acid sequence-based analysis for selected targets in the *P. aeruginosa* strains LESB58, PA14, PA7, 2192, C3719 and PACS2 as well as in *B. cenocepacia* J2315 and *E. coli* K12, and identification of homologs in humans. The percentage of protein sequence identity is indicated. A score of zero indicates no homologous sequences.

| **Locus tag** | **gene name** | **LESB58** | **PA14** | **PA7** | **% ID**    **2192** | | **C3719** | | **PACS2** | ***Bcc* J2315** | ***Ec* K12** | **Human**  **homologs** |
| --- | --- | --- | --- | --- | --- | --- | --- | --- | --- | --- | --- | --- |
| **PA0082** | *tssA1* | 98 | 98 | 90 | | 99 | | 98 | 97 | 27 | 29.7 | - |
| **PA0337** | *ptsP* | 100 | 100 | 99 | | 100 | | 100 | 100 | 35 | 42 | - |
| **PA0366** |  | 100 | 99 | 98 | | 100 | | 100 | 100 | 51 | 29 | - |
| **PA0396** | *pilU* | 100 | 99 | 99 | | 99 | | 100 | 100 | 33 | 37 | - |
| **PA0425** | *mexA* | 100 | 100 | 99 | | 100 | | 99 | 100 | 70 | 55 | - |
| **PA0534** | *pauB1* | 100 | 99 | 96 | | 100 | | 99 | 99 | 67 | 40 | - |
| **PA0593** | *pdxA* | 99 | 99 | 95 | | 99 | | 99 | 99 | 54 | 63 | - |
| **PA0823** |  | 98 | 98 | 30 | | 37 | | 32 | 98 | 31 | 34 | - |
| **PA0844** | *plcH* | 99 | 99 | 96 | | 99 | | 99 | 99 | 42 | 32 | - |
| **PA0914** |  | 99 | 99 | 78 | | 99 | | 99 | 98 | UH | 44 | - |
| **PA0996** | *pqsA* | 100 | 99 | 86 | | 100 | | 100 | 100 | 29 | 28.3 | 17 |
| **PA0998** | *pqsC* | 100 | 100 | 97 | | 100 | | 100 | 100 | 27 | 50 | - |
| **PA1030/P8** | *P8* |  |  |  | |  | |  |  |  |  |  |
| **PA1118** |  | 99 | 97 | 86 | | 98 | | 100 | 98 | 0 | 41 | - |
| **PA1523** | *xdhB* | 99 | 99 | 98 | | 99 | | 99 | 99 | 55 | 25 | 2 |
| **PA1542** |  | 100 | 100 | 95 | | 100 | | 100 | 100 | 44 | 27 | - |
| **PA1634** | *kdpB* | 99 | 99 | 98 | | 99 | | 100 | 99 | 60 | 60.6 | 23 |
| **PA1799** | *parR* | 100 | 99 | 94 | | 100 | | 100 | 100 | 50 | 42 | - |
| **PA2130** | *cupA3* | 99 | 99 | 69 | | 99 | | 98 | 99 | 27 | 31 | - |
| **PA2240** | *pslJ* | 100 | 100 | 99 | | 100 | | 100 | 100 | UH | 26 | - |
| **PA2385** | *pvdQ* | 99 | 99 | 94 | | 99 | | 99 | 99 | UH | 32 | - |
| **PA2414** |  | 99 | 99 | 94 | | 99 | | 99 | 99 | 25 | 41.4 | - |
| **PA2781** |  | 99 | 99 | 97 | | 92 | | 99 | 99 | 29 | 39 | - |
| **PA2854** |  | 100 | 100 | 98 | | 100 | | 100 | 100 | UH | 40 | - |
| **PA2873** | *tgpA* | 99 | 99 | 88 | | 98 | | 98 | 98 | UH | 48.7 | - |
| **PA2998** | *nqrB* | 100 | 100 | 99 | | 100 | | 100 | 100 | UH | 29.1 | - |
| **PA3058** | *pelG* | 100 | 99 | 96 | | 100 | | 100 | 99 | UH | 29 | - |
| **PA3071** |  | 100 | 99 | 96 | | 100 | | 100 | 100 | 32 | 38 | - |
| **PA3101** | *xcpT* | 99 | 98 | 99 | | 99 | | 99 | 99 | 63 | 55 | - |
| **PA3110** |  | 99 | 96 | 94 | | 64 | | 99 | 100 | UH | 26.1 | - |
| **PA3139** |  | 100 | 99 | 97 | | 100 | | 100 | 100 | 73 | 52 | 3 |
| **PA3257** | *prc* | 100 | 100 | 99 | | 100 | | 100 | 100 | 31 | 36 | - |
| **PA3270** |  | 99 | 100 | 95 | | 100 | | 100 | 100 | 37 | 30 | - |
| **PA3327** |  | 99 | 99 | 34 | | 99 | | 99 | 99 | 32 | 35 | - |
| **PA3448** |  | 99 | 99 | 98 | | 100 | | 100 | 100 | 66 | 43.1 | - |
| **PA3449** |  | 99 | 99 | 97 | | 99 | | 99 | 99 | 63 | 31 | - |
| **PA3460** |  | 100 | 100 | 98 | | 100 | | 99 | 100 | UH | 44 | - |
| **PA3493** |  | 100 | 100 | 86 | | 100 | | 94 | 100 | 0 | 46 | - |
| **PA3613** |  | 99 | 99 | 97 | | 100 | | 100 | 100 | 0 | 43 | - |
| **PA3622** | *rpoS* | 100 | 100 | 99 | | 100 | | UH | 100 | 46 | 76 | - |
| **PA3649** | *mucP* | 99 | 99 | 97 | | 99 | | 100 | 99 | 41 | 46 | - |
| **PA3761** | *nagE* | 99 | 99 | 94 | | 99 | | 99 | 99 | 55 | 47 | - |
| **PA3799** |  | 99 | 99 | 100 | | 100 | | 100 | 100 | 55 | 59 | 1 |
| **PA3867** |  | 39 | 98 | 96 | | 97 | | 40 | 36 | UH | 36 | - |
| **PA3950** |  | 100 | 99 | 95 | | 94 | | 99 | 99 | 70 | 51 | 42 |
| **PA4000** | *rlpA* | 99 | 99 | 97 | | 99 | | 89 | 99 | UH | 38.7 | - |
| **PA4059** |  | 99 | 98 | 96 | | 98 | | 97 | 98 | UH | 62 | 1 |
| **PA4098** |  | 100 | 100 | 96 | | 99 | | 100 | 100 | 63 | 34 | - |
| **PA4113** |  | 99 | 99 | 95 | | 99 | | 99 | 99 | 31 | 58 | - |
| **PA4116** | *bphO* | 99 | 99 | 85 | | 98 | | 100 | 100 | UH | 42 | - |
| **PA4265** | *tufA* | 100 | 100 | 100 | | 31 | | 31 | 100 | 82 | 84 | 17 |
| **PA4282** |  | 99 | 98 | 81 | | 98 | | 99 | 99 | UH | 28 | - |
| **PA4352** |  | 100 | 100 | 98 | | 100 | | 100 | 100 | UH | 22 | - |
| **PA4448** | *hisD* | 100 | 100 | 98 | | 100 | | 99 | 100 | 69 | 41 | - |
| **PA4489** | *magD* | 99 | 99 | 98 | | 99 | | 99 | 99 | UH | 53.3 | 1 |
| **PA4498** |  | 99 | 99 | 90 | | 86 | | 99 | 99 | 33 | 34 | - |
| **PA4515** |  | 99 | 99 | 96 | | 99 | | 99 | 99 | 58 | 49 | - |
| **PA4528** | *PilD* | 99 | 98 | 95 | | 99 | | 99 | 99 | 49 | 41.2 | - |
| **PA4684** |  | 99 | 100 | 99 | | 99 | | 99 | 100 | UH | 36 | - |
| **PA4767-68** |  |  |  |  | |  | |  |  |  |  |  |
| **PA4768** | *smpB* | 100 | 100 | 100 | | 100 | | 100 | 100 | 58 | 59 | - |
| **PA4809** | *fdhE* | 99 | 99 | 98 | | 99 | | 99 | 99 | 38 | 39 | - |
| **PA4916** |  | 99 | 100 | 98 | | 99 | | 99 | 99 | 0 | 31 | - |
| **PA5022** |  | 100 | 99 | 96 | | 99 | | 100 | 99 | 0 | 39 | - |
| **PA5138** |  | 100 | 100 | 98 | | 100 | | 100 | 100 | 0 | 56 | - |
| **PA5156** |  | 99 | 99 | 94 | | 99 | | 98 | 99 | 26 | 37 | - |
| **PA5202-03** |  |  |  |  | |  | |  |  |  |  |  |
| **PA5203** | *gshA* | 99 | 99 | 96 | | 99 | | 99 | 99 | 53 | 42 | - |
| **PA5332** | *crc* | 100 | 100 | 99 | | 100 | | 100 | 100 | 40 | 32.6 | 1 |
| **PA5360-61** | *phoB-phoR* |  |  |  | |  | |  |  |  |  |  |
| **PA5361** | *phoR* | 99 | 99 | 99 | | 99 | | 100 | 100 | 41 | 40 | - |
| **PA5548** |  | 99 | 98 | 28 | | 99 | | 99 | 98 | 31 | 23 | - |

UH	uncertain homolog
